# Bed side teaching: Student’s perception and its correlation with academic performance

**DOI:** 10.12669/pjms.36.6.2120

**Published:** 2020

**Authors:** Shahid Sarwar, Abdul Aleem, Muhammad Arif Nadeem

**Affiliations:** 1 Dr. Shahid Sarwar, MBBS, FCPS (Med), FCPS (Gastroenterol) MCPS-HPE, FRCP (Edin). Associate Professor, Medical Unit III, Department of Medicine and Gastroenterology, Services Institute of Medical Sciences Lahore, Lahore - Pakistan; 2 Dr. Abdul Aleem, MBBS. Post Graduate Resident Gastroenterology, Medical Unit III, Department of Medicine and Gastroenterology, Services Institute of Medical Sciences Lahore, Lahore - Pakistan; 3 Dr. Muhammad Arif Nadeem, MBBS, FCPS (Med). Professor of Medicine & Gastroenterology, Medical Unit III, Department of Medicine and Gastroenterology, Services Institute of Medical Sciences Lahore, Lahore - Pakistan

**Keywords:** Academic performance, Bedside teaching, Student’s perception

## Abstract

**Objective:**

To determine student’s perception of bedside clinical teaching and to correlate it with their performance in assessment.

**Methods:**

This cross-sectional study of correlational survey was conducted at Services Institute of Medical Sciences in September 2019, involving students of final professional year who filled a proforma to rate their bedside teaching experience during clinical rotations using rating scale. Mean scores of items were determined with score < 3 reflecting dis-satisfaction. Mean scores were compared between high and low performing students using student’s t test.

**Results:**

Total of 160 students participated. Physical environment domain was assigned lowest scores by students (mean 2.94±0.74) followed by teaching task by teachers (3.04±0.72), group dynamics (3.16±0.81) and patient comfort and attitude towards patient (3.87±0.60). Teaching task by teacher had maximum stems with scores < 3 needing significant improvement. Students with low academic performance were more unsatisfied with group dynamics of bedside teaching (p value 0.009), especially lack of equal opportunities of participation for every member (p value <0.000) in clinical rotations.

**Conclusion:**

Small size group with adequate space for bedside training and faculty training can enhance learning experience of students. Ensuring active participation of each group member during bedside learning can improve academic performance of students.

## INTRODUCTION

Educational philosophy, over last few decades has moved from behaviorism i.e. teacher centered approach to constructivism i.e. learner centered education.^1^ Essence of curriculum is to develop a learner who knows how to learn rather than passive transfer of knowledge and skills.^2^ This shift in paradigm has introduced newer instructional tools like small group discussion, problem based learning, simulations, peer assisted learning etc. Space for traditional tools of teaching like lectures in curricular plans of modern medical institutions is fast shrinking.^3^

This transformation however is very slow in our undergraduate medical curriculum. We are still dependent on lectures, seminars and tutorials as major tools for knowledge transfer. Due to lack of proper infrastructure, time constrains, insufficient faculty training, newer learner centered tools are still not being effectively used in our teaching programs.^4^ Clinical skill training that includes comprehensive clinical interviewing, performing clinical examination and developing management plan is carried out predominantly via bed side teaching.

Although our resources in terms of infrastructure, equipment and faculty are limited, especially in public sector medical colleges but as our hospital are over-crowded with patients, we have no shortage of patients and diversity of clinical presentations. Therefore, bedside teaching if used appropriately can compensate for absence of modern innovative tools for skill training in our hospitals.^5^

Bedside teaching is a true contextual learning where students have opportunity to interact and examine patients and solve clinical questions in real time. They have first-hand experience of what they are expected to do in practical life. However as this learning takes place in wards where critical patients are being managed, ward rounds are taking place, diagnostic as well as therapeutic interventions are being done therefore learning environment is difficult to control. Learner has to learn in places populated by patients, their attendants, doctors, nurses and paramedics. It is the responsibility of tutor to make this environment conducive to learning in the presence of all these factors otherwise learner can easily get distracted and disengaged.^6^

Bedside teaching being our major tool for clinical skill training, we need to determine whether it is being effectively used and is our learner satisfied with his training in these sessions. Moreover, it will be interesting to see whether learner satisfaction with bedside teaching reflects in his performance in assessment or not. This information can be used to inform our faculty to make bed side teaching sessions more effective and conducive to learning.^7^

We planned a study to determine perception of our students regarding their bedside teaching in final professional year and to correlate this perception with their performance in summative assessment.

## METHODS

This cross sectional study for correlational survey was conducted at Services Institute of Medical Sciences (SIMS), Pakistan in September 2019, last month of academic calendar of college. Approval was obtained from the institutional review board (Ref.No. IRB/2019/600/SIMS, Dated December 12, 2019). Purposive non-probability sampling was done by only including students of final professional year of MBBS who had participated in at least three of four clinical rotations and its test during academic year. Informed consent was taken from the participants. Students not willing to participate and those having appeared in only two or less ward tests were excluded. Students filled the study proforma in a dedicated session of 30 minutes regarding their perception of bedside teaching experienced during clinical rotations after brief instructions by researcher.

Instrument used for evaluation of bedside teaching was a validated structured self-administered questionnaire with a five point Likert scale ranging from strongly disagree to strongly agree (scored as 1-strongly disagree, 2-disagree, 3 unsure, 4-agree and 5-strongly agree) to be used for obtaining information from the students. The questionnaire had four domains, (i) physical environment of bedside sessions, (ii) patient’s comfort and attitude towards patient, (iii) teaching tasks of teacher and (iv) group dynamics. There were 5 items in physical environment domain, 7 in patient’s comfort, 9 in teaching tasks and 4 in group dynamics domain. Scores for these domains were calculated by determining mean of scores of its items which varied from 1 to 5.

Academic performance in clinical rotations was determined by calculating mean of percentage marks obtained in all clinical ward tests by students. Students with percentage of 71% or above were classified as high performers while those with less than 71% percent mean score were labeled as low performers.

### Statistical Analysis

Data was analyzed by using SPSS^®^22. Response of students for items were scored from 1 to 5 depending on their selection from strongly disagree to strongly agree. For each item and 4 domains of proforma, mean score and standard deviation (SD) were calculated. Interpretation of mean scores (annex-II) was as follows-

5= No need of further improvement

4 to <5= Very minimum efforts are needed to fulfill the required criteria.

3 to <4= Some efforts are needed to fulfill the required criteria.

2 to <3 = Moderate efforts are needed to fulfill the required criteria.

1 to <2= Considerable efforts are needed to fulfill the required criteria.

Mean score less than three was considered sub-optimal. Mean scores in each item and in four domains of proforma were compared between high performing and low performing students using unpaired student’s *t test* to confirm or refute hypothesis that student’s perception regarding bedside teaching does affect his academic performance.

## RESULTS

Out of 180 enrolled students of final year MBBS, 160 meeting inclusion criteria participated in survey. Male to female ratio in participants was 1/2.8 (42/118). Out of four domains surveyed, patient’s comfort and attitude towards patient had best mean score of 3.87 (standard deviation ±0.6) (range 2.1-5) thus needing some effort to improve it further while mean scores for teaching task of teachers, 3.04 (±0.72) and group dynamics of class, 3.16(±0.81) highlights significant room for improvement. Low mean score of 2.94(±0.74) of physical environment reflected student’s dissatisfaction with their learning environment. Detailed mean scores for each stem of survey are shown in Table-I.

**Table-I:** Mean scores for each stem of survey.

Survey Stem	Mean score± (Standard deviation SD)
***Physical environment***	
There was comfortable temperature	3.14 (±1.24)
There was no disturbance by noise	2.86 (±1.1)
There was sufficient light	3.69 (±0.92)
There was adequate space to stand and observe all activities with the patient	2.38 (±1.10)
Student number was adequate so everyone had chance to participate	2.61 (±1.12)
***Patient’s comfort and attitude towards patient***	
Informed consent of patient	4.38 (±0.72)
I was introduced to patient properly	4.17 (±0.89)
We maintained privacy of patient	3.62 (±1.10)
All findings were explained to patient	2.91 (±1.2)
We responded to patient’s questions	3.78 (±0.72)
We were sympathetic to patient and paid attention to his comfort and emotions	3.93 (±0.81)
We thanked patient for his participation	4.24 (±0.62)
***Teaching task of teachers***	
Patient was selected ahead of patient	2.78 (±1.14)
Teacher observed us during interview of patient	2.84 (±1.19)
Teacher observed us during clinical examination	3.34 (±1.1)
I had adequate scope to practice skills	2.99 (±1.13)
Teacher always assisted me in skill practice	2.97 (±1.14)
Teacher encouraged us to think during discussion	3.97 (±0.70)
Constructive feedback was given by teacher	2.94 (±1.02)
Teacher summarized the session effectively	3.15 (±1.05)
Teacher started and finished class on time	2.49 (±1.17)
***Group Dynamics of the class***	
I was clear about our group role in learning	3.03 (±1.09)
I was clear of my role right from beginning	3.15 (±1.07)
I actively participated throughout the class	3.56 (±1.02)
We have finished every task in time	2.91 (±1.1)

Aspects of bedside teaching in need of major improvement as perceived by students with mean score < 3 were noisy environment (mean score 2.86±1.1), lack of adequate space for students to observe activities (mean score 2.38±1.1), more number of students in group (mean score 2.61±1.1), failure to explain findings to patient (mean score 2.91±1.2), failure of teachers to select patient for session ahead of time (mean score 2.78±1.1), lack of direct supervision during patient’s interview (mean score 2.84±1.1), inadequate opportunities to practice skills (mean score 2.99±1.1), insufficient assistance of teacher in skill practice (mean score 2.97±1.1), absence of constructive feedback (mean score 2.94±1.02), failure to complete session in time (mean score 2.49±1.1) and inability of group of students to complete task in time (mean score 2.91±1.1).

We compared mean scores for four domains of bedside teaching between male and female students as shown in Table-II. Despite no significant difference, relatively lower scores in female students emphasizes need for special attention to their learning needs.

**Table-II:** Comparison of perception regarding bedside teaching between male and female students.

Domains of bedside teaching	Male (N-42) Mean ± SD	Female (N-118) Mean ± SD	P value
Physical environment	3.11 (0.77)	2.89 (0.73)	0.1
Patient comfort and attitude towards patient	3.94 (0.63)	3.84 (0.59)	0.37
Teaching task by teacher	3.20 (0.76)	2.99 (0.70)	0.1
Group dynamics of group	3.23 (0.98)	3.13 (0.07)	0.46
Total mean score of four domains	3.37 (0.58)	3.21 (0.47)	0.07

In order to correlate student’s perception regarding quality of bedside teaching with his performance in assessment, we compared the mean scores of high and low performing students in four domains of bedside teaching for 141 students with available results of at least three ward tests as shown in Table-III. Both groups had comparable perception regarding bedside sessions in all domains except group dynamics where low performing students had assigned significantly lower scores depicting their dissatisfaction (p value 0.009). On analysis of items of group dynamics domain, low performing students had rated significantly lower scores for equal opportunity of participation throughout the bedside session, mean score 3.22 vs 3.86 of high performers (p value 0.000).

**Table-III:** Comparison of low performing and high performing students for perception regarding bedside teaching.

	Low performing students (n-51)	High performing students (n-90)	P value
Physical environment	3.05 (0.73)	2.89 (0.77)	0.22
Patient comfort and attitude towards patient	3.78 (0.61)	3.91 (0.61)	0.23
Teaching task of teacher	3.04 (0.72)	3.11 (0.70)	0.61
Group dynamics	2.97 (0.92)	3.34 (0.70)	0.009
I was clear about group role	2.88 (1.1)	3.17 (1.07)	0.14
I was clear about my role right from start	3.02 (1.1)	3.32 (1.03)	0.10
I actively participated throughout the class	3.22 (1.08)	3.86 (0.85)	0.000
We have finished every task in time	2.8 (1.13)	3.02 (1.13)	0.27

Total score	3.21 (0.55)	3.31 (0.47)	0.26

## DISCUSSION

A renowned clinician-teacher, Sir William Osler stated in 1903 “To study the phenomena of disease without a book is to sail an uncharted sea, whilst to study books without patients is not to go to sea at all”.^8^ While interacting with patients, student have an opportunity to learn communication skills, clinical examination skills, humanism, empathy and professionalism. Efficient utilization of bedside teaching and identification of its limitations needs feedback from its main stakeholders, i.e. students.

In last few years, due to increasing accountability, more patient autonomy, issues of patient’s privacy, focus on competency based clinical teaching and evolving knowledge of how students learn, bedside teaching practices have remarkably changed all over the world.^9^ Introduction of web based learning, case based learning (CBL) and integrated teaching models has transformed bedside training. However continued presence of factors like time constrains, pressure of clinical and administrative work on teachers, declining bedside teaching skills and dependence on technology like simulation can compromise optimum utilization of this learning opportunity.^10^,^11^ Ahmed have noticed that due to lack of intellectual excitement and mediocre teaching skills practiced in bedside teaching sessions, clinical skills of doctors are fast declining.^12^ Only real time evaluation of beside teaching to identify areas in need for improvement can ensure training of competent professionals.

In our survey, physical environment domain received lowest scores by students. They have identified noisy environment, lack of adequate space for students to stand and observe activities and more number of students in a batch as major physical barriers in bedside teaching. In a study by Amir N et al mean scores for physical environment were 2.95, 3.32 and 3.42 for 3^rd^, 4^th^ and 5^th^ year students of MBBS.^13^ In another study, 63.23% students regarded more number of student in group as major factor hampering their learning.^14^ Nandini C et al identified large size group of students and lack of physical space due to overcrowding as limiting factors highlighted by students during their clinical training.^15^ Between 2 to 5 is considered most appropriate number of participants for bedside clinical activity.^16^

Highest mean score among 4 domains was for patient’s comfort and attitude towards patient (3.87) and only stem with less than three score was failure to explain findings to patient (2.91). Similar student’s feedback was noted by Sultana J et al.^17^ She believes that high scores in domain of student’s attitude towards patients depicts their inability to be impartial in self-evaluation.

Maximum number of stems with mean score less than three are related to teaching task of teacher whose mean score itself is 3.04. Students are not satisfied with their teachers because of failure to timely select patient for teaching (2.78), lack of direct supervision of patient’s interview (2.84), insufficient opportunities for skill practice (2.99), no assistance during skill practice (2.97), inability to give constructive feedback (2.94) and failure to complete session on time (2.49).

In a study by Jones P et al, only 45% students agreed that their bedside learning is adequately supervised.^18^ Jayakumar N noted that fluctuating health status of admitted patients and rapid turnover of patients can be a limiting factor in timely selection of patient for teaching.^10^ The optimal approach to address student’s dissatisfaction with opportunities to practice skills and lack of supervision will need students seeing patients on a one to one basis.^19^ Lack of feedback or poor quality of feedback by teacher was also observed in another study by Ramani S et al.^20^ A focus group study identified busy schedules of clinical teachers being responsible for student’s dissatisfaction with their training.^21^

Students with low scores in assessment were not satisfied with group dynamics of bedside teaching especially lack of equal opportunity for everyone to participate. As students of different intellectual abilities, learning styles and social behavior are included in a group, bedside teaching session is likely to be dominated by brilliant and self-confident students, depriving average or less competent students of opportunity to participate and learn. It is the duty of teacher to ensure equal participation of every member of group in interviewing, skill practice and discussion.^16^

In our study students gave their perception regarding bedside teaching during their rotations in different specialties over one academic year. Factors like halo effect where one bad experience can cloud student’s opinion and recall bias may compromise their perception. Similarly, apart from his satisfaction with bedside teaching environment, group dynamics and teacher’s effort, his hard work, level of intelligence and learning style are also important determinants of his academic performance and these factors should be kept in perspective while evaluating his assessment results. Moreover, data based in one institution may be difficult to generalize for other teaching centers.

Despite these limitations we can infer that focus on faculty development, reducing clinical and administrative responsibilities of teachers to give protected time for teaching, providing facilities like dedicated room for teaching and regular monitoring of bedside teaching sessions by institutions can improve quality of bedside teaching. Moreover, lesser number of students in a group and active engagement of every member in teaching sessions can enhance this learning experience.

## CONCLUSION

Small size group with adequate space for conducting bedside training along with faculty training to create awareness of their responsibilities can enhance learning experience of students. Ensuring active participation of all group members during learning can improve academic performance of students.

### Author’s Contribution:

**SS:** Conception and design, acquisition of data, analysis and interpretation, drafting of article, approval of the version, agreement to be accountable for all aspect

**AA:** Acquisition of data, revising the manuscript, approval of the version, agreement to be accountable for all aspect

**MAN:** Conception and design, revising manuscript critically, final approval of the version and agreement to be accountable.
